# Supplementary material for: Photoinduced hydrogen dissociation in thymine predicted by coupled cluster theory
Source: Nat Commun. 2024 Nov 22;15:10128. doi: 10.1038/s41467-024-54436-2 (PMC11584849; doi:10.1038/s41467-024-54436-2)
Supplement: Supplementary file 1 — Supplementary information [file 41467_2024_54436_MOESM1_ESM.pdf]

Supplementary notes for "Photoinduced hydrogen dissociation in thymine predicted by coupled cluster theory"

Eirik F. Kjønstad*[1,2,3], O. Jonathan Fajen[1,2], Alexander C. Paul[3], Sara Angelico[3], Dennis Mayer[4], Markus Gühr[4,5], Thomas J. A. Wolf[2], Todd J. Martínez*[1,2], Henrik Koch*[3]

[1]Department of Chemistry, Stanford University, Stanford, CA, USA.
[2]Stanford PULSE Institute, SLAC National Accelerator Laboratory, Menlo Park, CA, USA.
[3]Department of Chemistry, Norwegian University of Science and Technology, Trondheim, 7491, Norway.
[4]Deutsches Elektronen-Synchrotron DESY, Hamburg, Germany.
[5]Institute of Physical Chemistry, University of Hamburg, Hamburg, Germany.

Contributing authors: eirik.kjonstad@ntnu.no; todd.martinez@stanford.edu; henrik.koch@ntnu.no;

Contents

1 Estimation of time constants: the rate of $\pi\pi^*$ decay and of $\pi\pi^*/n\pi^*$ internal conversion S3

1.1 Adiabatic populations and time constants S3
1.2 Diabatic populations and time constants S4
1.3 Time constants from simulated oxygen-edge X-ray absorption spectrum S9

2 Simulated oxygen-edge X-ray absorption spectrum S11

3 Initial dynamics for one initial condition with aug-cc-pVDZ S19

4 Details on initial conditions that end in N-H dissociation S20

5 Simulated UV-vis absorption spectrum S24

6 Inclusion of S_3 in dissociative initial conditions S27

7 Potential energy curves along the NH bond with CC3 and aug-cc-pVDZ S28

8 Nitrogen-edge spectrum at Franck-Condon, at the $n\pi^*$ minimum, and at $Q = 1.0$ in the dissociative N_1-H pathway S30

9 Wigner sampling: selection of initial conditions S32

10 Spawning geometries and the accessibility of the conical intersection seam along the C_5-C_6 stretching coordinate S33

11 Stationary points on the excited state surfaces: the minimum energy S_1/S_2 conical intersection and the S_1 minimum S34

12 Interface for AIMS dynamics with CC theory S36

Suppl. Note 1

Estimation of time constants: the rate of $\pi\pi^*$ decay and of $\pi\pi^*/n\pi^*$ internal conversion

In order to model the kinetics of $\pi\pi^*$ decay and the $\pi\pi^*/n\pi^*$ internal conversion, we first assume that the $\pi\pi^*$ state is populated at time $t = 0$, that is, $P_{\pi\pi^*}(0) = 1$ and $P_{n\pi^*}(0) = 0$. We further assume a partial transfer of population from $\pi\pi^*$ to $n\pi^*$ that decays exponentially. In the simulation, all initial conditions begin on the $\pi\pi^*$ state and the majority of them become trapped in the $n\pi^*$ state, consistent with the above two assumptions. In more detail, we have

$$P_{\pi\pi^*}(t) = 1 - C_2(1 - \exp(-t/\tau_{\pi\pi^*})) \quad (1)$$

$$P_{n\pi^*}(t) = C_1(1 - \exp(-t/\tau_{n\pi^*})). \quad (2)$$

The time constants $\tau_{\pi\pi^*}$ and $\tau_{n\pi^*}$ give the characteristic time for $\pi\pi^*$ decay and the rate of $\pi\pi^*/n\pi^*$ conversion, respectively.

This simple model does not give a complete picture of the observed dynamics. In the simulation, we find two initial conditions that terminate at an S_1/S_0 intersection. Hence, there is a second decay channel (in particular, $\pi\pi^*$ to $\pi\sigma^*$). The proportion of dissociative decay is assumed to be sufficiently small to be neglected in the models. This should be a good approximation since only a small number of conditions participate in the channel (2/16). In the population statistics below, we assume that these two conditions decay instantly to S_0 when they are terminated.

1.1 Adiabatic populations and time constants

An initial estimate of the $\pi\pi^*/n\pi^*$ internal conversion time constant $\tau_{n\pi^*}$ and the $\pi\pi^*$ lifetime $\tau_{\pi\pi^*}$ can be extracted directly from the adiabatic populations. We here assume that S_1 is always of $n\pi^*$ character, and S_2 of $\pi\pi^*$ character, that is,

$$P_{S_2}(t) = P_{\pi\pi^*}(t) \quad (3)$$

$$P_{S_1}(t) = P_{n\pi^*}(t) \quad (4)$$

The adiabatic populations, along with time constants, are shown in Suppl. Fig. 1. In terms of the adiabats, we find $\tau_{\pi\pi^*} = 18 \pm 1\,\mathrm{fs}$ and $\tau_{n\pi^*} = 17 \pm 1\,\mathrm{fs}$. These estimated time constants are too low, as we can show by considering the character of the wavepacket.

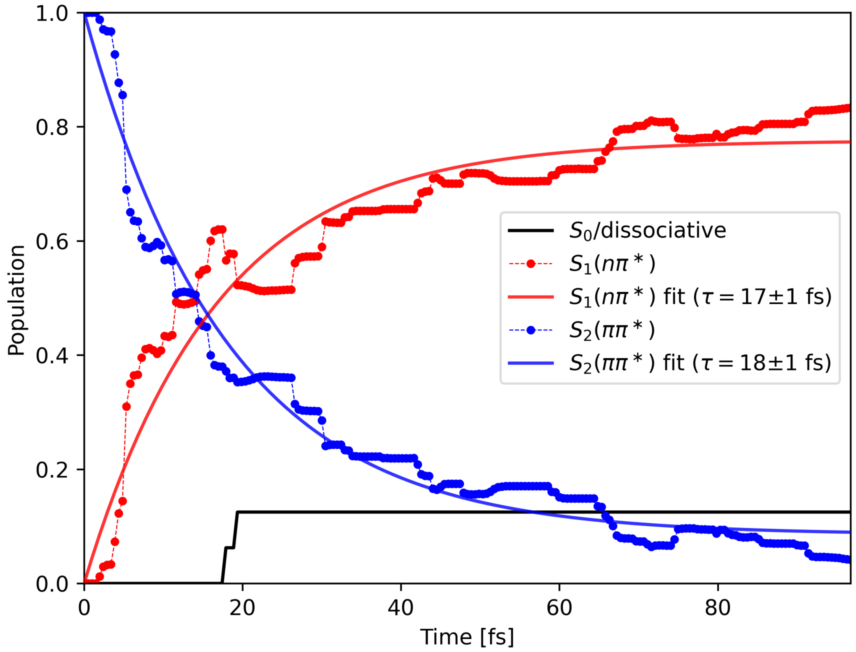

Suppl. Fig. 1 : Adiabatic poopulations for S_1 and S_2 with associated lifetimes. For every number of initial conditions included in the fitting, the error bars on the characteristic time τ are reported as two standard deviations. The two dissociative initial conditions terminate at around 20 fs and are here taken to transfer completely to S_0 through the dissociative channel.

1.2 Diabatic populations and time constants

To find more accurate estimates, we can reassign the populations according to the state character of the adiabatic states, thereby directly estimating $P_{\pi\pi^*}(t)$ and $P_{n\pi^*}(t)$. To calculate the contribution from a populated adiabatic state to the constituent diabatic states, $\pi\pi^*$ and $n\pi^*$, we assume that the electronic transitions from the ground state to each of the diabats remain well-defined as bright and dark, respectively. Given that the deviations from planarity are generally small, this should be a good approximation. For a populated adiabatic state, S_k, we assign the population to $\pi\pi^*$ and $n\pi^*$ according to the oscillator strengths f_{if} (i = initial, f = final). In particular, if $k = 2$ (normally $\pi\pi^*$), then

$$P^k_{\pi\pi^*} = \frac{\max(f_{01}, f_{02})}{f_{\text{tot}}}, \quad P^k_{n\pi^*} = 1 - P^k_{\pi\pi^*}, \tag{5}$$

where $f_{\mathrm{tot}} = f_{01} + f_{02}$, and if $k = 1$ (normally $n\pi^*$), then

$$P^k_{n\pi^*} = 1 - \frac{\min(f_{01}, f_{02})}{f_{\mathrm{tot}}}, \quad P^k_{\pi\pi^*} = 1 - P^k_{n\pi^*}. \tag{6}$$

This procedure usually results in a clear assignment, with one adiabatic state accounting for $\geq 99\%$ of the total brightness. In the vicinity of the S_1/S_2 intersection, however, S_1 and S_2 assume more mixed electronic characters, and the reassigned population is more evenly shared between the diabats. Calculated diabatic populations, along with estimated time constants, are shown in Suppl. Fig. 2. From the diabatic populations, we find that the time constants for conversion are

$$\tau_{\pi\pi^*} = 34 \pm 6\,\mathrm{fs} \tag{7}$$
$$\tau_{n\pi^*} = 37 \pm 9\,\mathrm{fs}, \tag{8}$$

where error bars are given as two standard deviations. Clearly, the $\pi\pi^*$ to $n\pi^*$ transfer is slower than expected from the adiabats. Notice that at short and long times, the adiabatic and diabatic populations agree, indicating a clear separation of $n\pi^*$ and $\pi\pi^*$ character close to the Franck-Condon point and close to the $n\pi^*$ minimum. At intermediate times (around 10 fs to 50 fs), state-mixing prevents a clear, binary assignment of the adiabats. Furthermore, although transfer to S_1 occurs rapidly from 10 fs to 30 fs, this is not accompanied by a similar change from $\pi\pi^*$ to $n\pi^*$ character. The less rapid population transfer of the diabats, as compared to the adiabats, is consistent with the slower appearance of the $n\pi^*$ signal in the simulated spectrum, as can be seen from Figure 2. This fact is discussed further in Section 1.3.

Indeed, there are multiple instances of population transfer from S_2 to S_1 in which the electronic state retains $\pi\pi^*$ character for 10-15 fs. These $S_1/\pi\pi^*$ trajectories demonstrate unique behavior in the C_5–C_6 and C_4–O_8 plane, as seen in Suppl. Fig. 3. The trajectories on S_1 with $\pi\pi^*$ character move toward longer C_5–C_6 and C_4–O_8 distances before turning around and moving toward the much shorter C_5–C_6 distance and longer C_4–O_8 characterizing the $n\pi^*$ minimum, acquiring $n\pi^*$ character along the way. Trajectories that spawn on S_1 with $n\pi^*$ character, on the other hand, move directly toward longer C_4–O_8 and shorter C_5–C_6 distances.

The error bars in $\tau_{\pi\pi^*}$ and $\tau_{n\pi^*}$ give the error of the fit and does not include any other source of error; for example, it does not account for the degree of convergence of the wavepacket dynamics. In Suppl. Fig. 4, we show the calculated diabatic time constants versus the number of included initial conditions (1 to 16). This gives some indication of the degree of convergence of the wavepacket dynamics. In particular, the changes in the constants settle at around 5 to 10 initial conditions, exhibiting relatively small changes after this point (on the order of a few to around five fs).

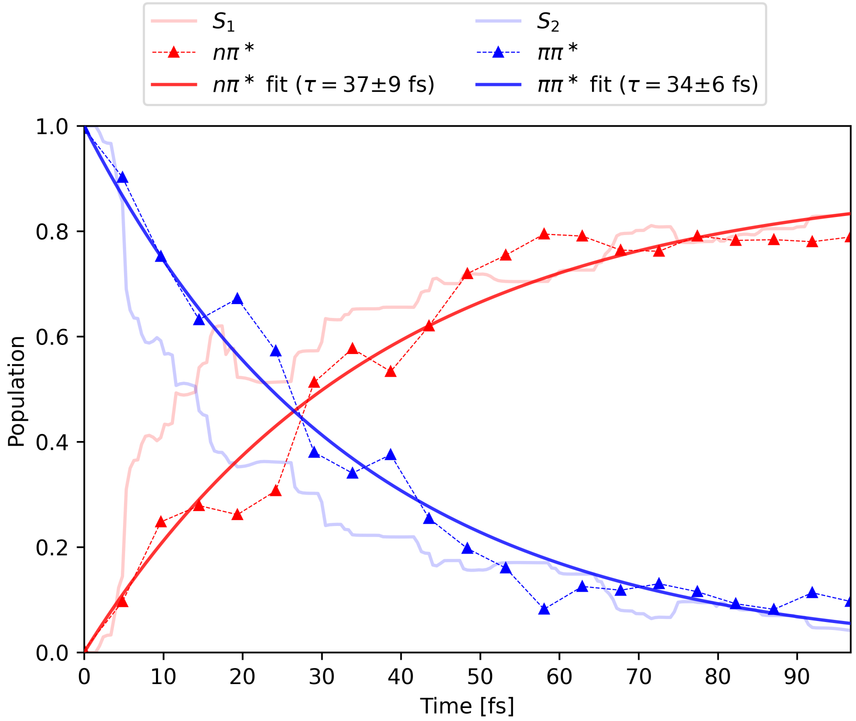

Suppl. Fig. 2 : Diabatic populations for $n\pi^*$ and $\pi\pi^*$ with associated lifetimes. The error bars on the characteristic time τ is reported as two standard deviations. The two dissociative initial conditions terminate at around 20 fs and are here taken to transfer completely to S_0 through the dissociative channel. The population in this channel is not shown. The adiabatic populations are shown in solid lines and illustrate the more rapid transfer of the adiabats as compared to the diabats.

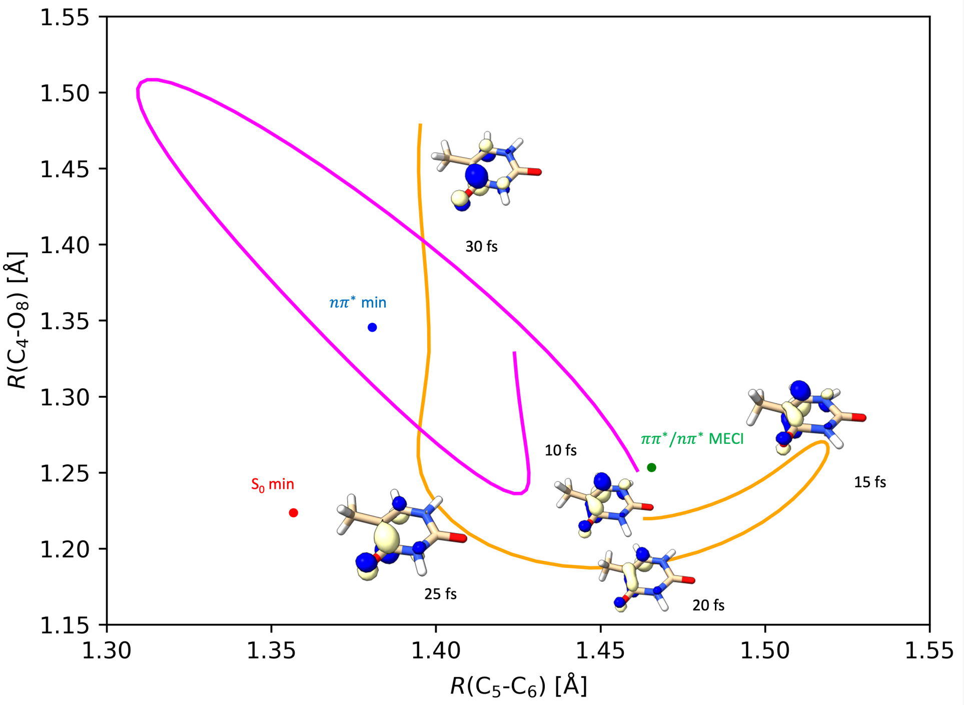

Suppl. Fig. 3 : A representative IC (IC 11) that features a population transfer from $S_2 - S_1$ retaining $\pi\pi^*$ character (orange) and a later transfer from $S_2 - S_1$ which changes from $\pi\pi^*$ to $n\pi^*$ (magenta). For the $S_1/\pi\pi^*$ trajectory, we visualize the dominant virtual NTO at intervals of 5 fs after spawn. Initially, the dominant virtual NTO corresponds to the π^* orbital of $\pi\pi^*$ state, but as the trajectory nears the $n\pi^*$ minimum, it corresponds to the π^* orbital of the $n\pi^*$ state. Compare with Figure 1 in main text.

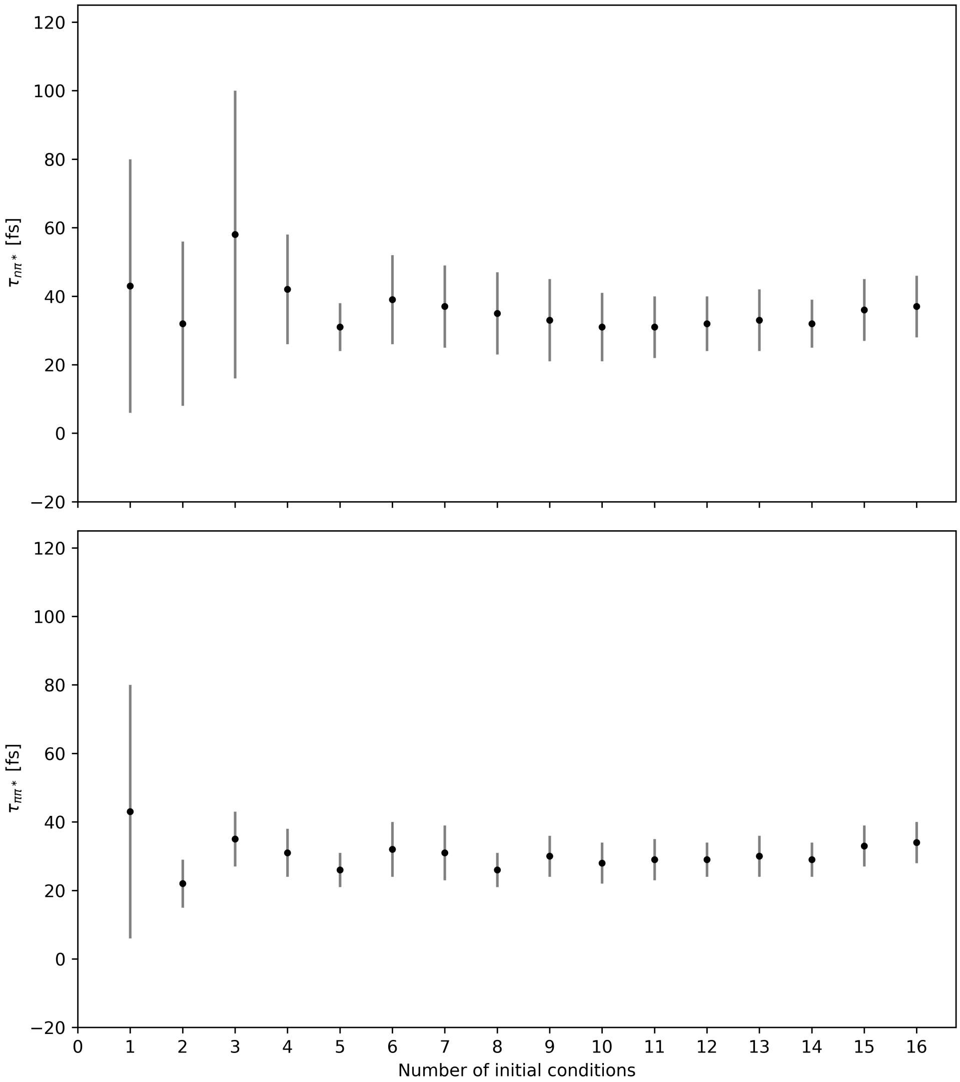

Suppl. Fig. 4 : Convergence of time constants calculated from diabatic populations. Upper panel: $\pi\pi^*/n\pi^*$ conversion. Lower panel: $\pi\pi^*$ decay. The error bars are given as two standard deviations.

1.3 Time constants from simulated oxygen-edge X-ray absorption spectrum

The feature at 526 eV is associated with the $n\pi^*$ state, as was previously indicated by calculations at the minimum on the S_1 surface [1]. In our simulated spectrum (Figure 2), we similarly find a feature at around 526 eV that grows in intensity in the first 50 fs to 70 fs. If the feature is in fact due to the $n\pi^*$ character, as the evidence suggests, then we expect the $n\pi^*$ population and the intensity of the peak to be correlated.

To compare the peak intensity (at various times) and the diabatic $n\pi^*$ population, we shift the intensities such that the $n\pi^*$ intensity is zero at $t = 0$ and such that the maximum $n\pi^*$ intensity is equal to the maximum diabatic population of the $n\pi^*$ state. This shifted and rescaled intensity will then give a measure of the $n\pi^*$ diabatic population, provided the signal is caused by the $n\pi^*$ character. Similarly, the $\pi\pi^*$ state has a feature at around 534 eV, which is masked by the ground state bleach in Figure 2. By the same argument, the intensity of this peak provides a measure of the population of the $\pi\pi^*$ state. We similarly rescale this $\pi\pi^*$ intensity so that the intensity is 1 at $t = 0$.

The peak intensities, along with diabatic and adiabatic populations, are compared in Suppl. Fig. 5. We see that the diabatic population of the $n\pi^*$ state closely follows the growth of the 526 eV signal; similarly, the decay of the $\pi\pi^*$ diabat follows the decay of the 534 eV signal. The time constants are also in agreement with the diabats: we find $\tau_{n\pi^*} = 41 \pm 14$ fs (from the intensity) versus $\tau_{n\pi^*} = 37 \pm 9$ fs (diabat) and $\tau_{\pi\pi^*} = 27 \pm 4$ fs (intensity) versus $\tau_{\pi\pi^*} = 34 \pm 6$ fs (diabat).

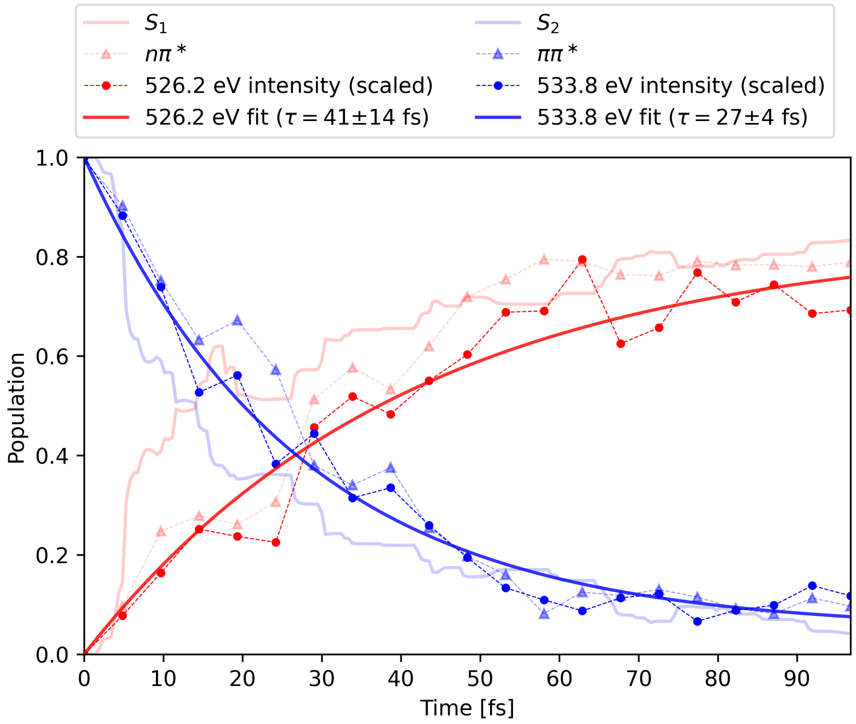

Suppl. Fig. 5 : Scaled intensities at 526.2 eV and 533.8 eV, as well as adiabatic populations for S_1 and S_2 and diabatic populations for $n\pi^*$ and $\pi\pi^*$ with associated lifetimes. The error bars on the characteristic time τ is reported as two standard deviations.

Suppl. Note 2

Simulated oxygen-edge X-ray absorption spectrum

The X-ray absorption of the excited states of thymine was calculated using a local development version of the eT program [2]. To compute the absorption strengths, we used CCSD/cc-pVDZ for the valence excited states and CC3/cc-pVDZ for the core excited states, where the core states were determined with the core-valence separation approximation [3]. Using CCSD for the valence states ensures that the valence excited states in the dynamics simulation match the valence excited states in the X-ray absorption calculations. From the CCSD and CC3 calculations, we define the energies

$$E(S_0) = E_{\mathrm{CCSD}}(S_0) \tag{9}$$

$$E(S_n^{\mathrm{val}}) = E_{\mathrm{CCSD}}(S_0) + \omega_{\mathrm{CCSD}}(S_n^{\mathrm{val}}) \tag{10}$$

$$E(S_n^{\mathrm{core}}) = E_{\mathrm{CC3}}(S_0) + \omega_{\mathrm{CC3}}(S_n^{\mathrm{core}}) \tag{11}$$

and the energy differences

$$E(S_n^{\mathrm{core}}) - E(S_n^{\mathrm{val}}) = \omega_{\mathrm{CC3}}(S_n^{\mathrm{core}}) - \omega_{\mathrm{CCSD}}(S_n^{\mathrm{val}}) + [E_{\mathrm{CC3}}(S_0) - E_{\mathrm{CCSD}}(S_0)] \tag{12}$$

$$E(S_n^{\mathrm{core}}) - E(S_0) = \omega_{\mathrm{CC3}}(S_n^{\mathrm{core}}) + [E_{\mathrm{CC3}}(S_0) - E_{\mathrm{CCSD}}(S_0)] \tag{13}$$

This produces an effective shift in both types of energy differences (valence-to-core, ground-state-to-core) equal to $E_{\mathrm{CC3}}(S_0) - E_{\mathrm{CCSD}}(S_0)$. Since we shift the spectra to align the ground state peak, and since the variation of $E_{\mathrm{CC3}}(S_0) - E_{\mathrm{CCSD}}(S_0)$ is small, this does not have a significant effect on the simulated spectra. All spectra were shifted by -0.5 eV to match the experimental ground state bleach at 531.4 eV [1].

In the first 800 au ($\sim$19.4 fs), we chose to calculate the absorption spectra at time steps of 20 au ($\sim$0.5 fs) to follow the decay of the $\pi\pi^*$ and the appearance of the $n\pi^*$ states more closely. From 800 au to 4000 au ($\sim$96.76 fs), a time step of 200 au ($\sim$4.84 fs) was chosen. To generate smooth spectra and match the experimental width, individual excitations were broadened using Gaussian functions with full width at half maximum (FWHM) of 0.3 eV (see Suppl. Fig. 6). For the false color plots, the data was additionally interpolated along the time axis. This was done using Gaussian interpolation as provided by `matplotlib`'s [4] `imshow` (see Suppl. Fig. 7 and Suppl. Fig. 8) or by broadening individual excitations with two-dimensional Gaussians with FWHM 0.3 eV along the energy axis and FWHM 10 fs to 70 fs along the time axis (see Suppl. Fig. 9, Suppl. Fig. 10, Suppl. Fig. 11, and Suppl. Fig. 12).

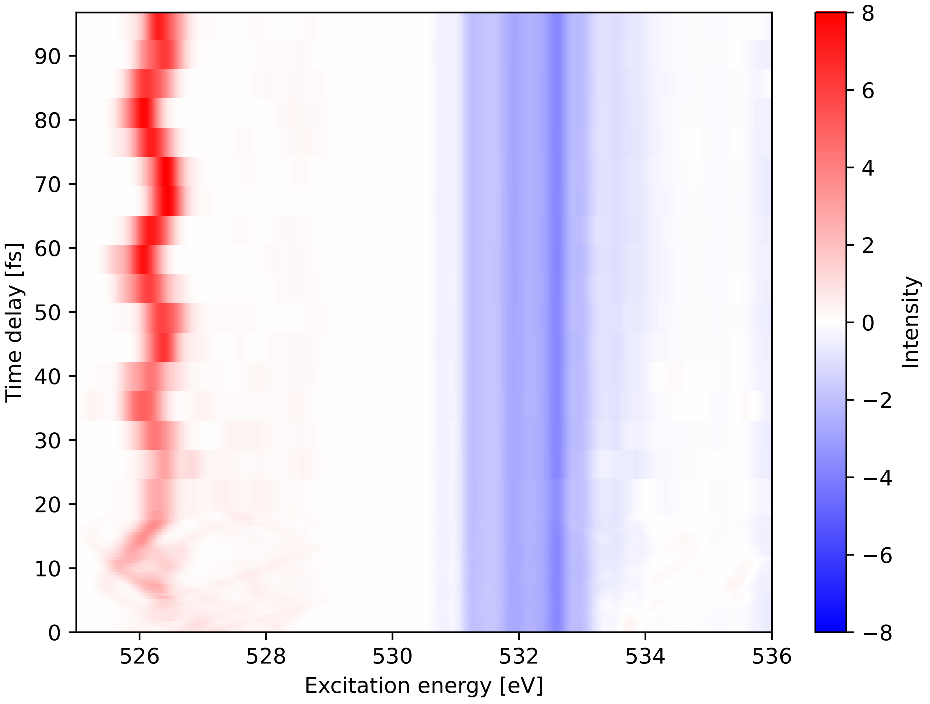

Suppl. Fig. 6 : False color plot without smoothing in the time direction.

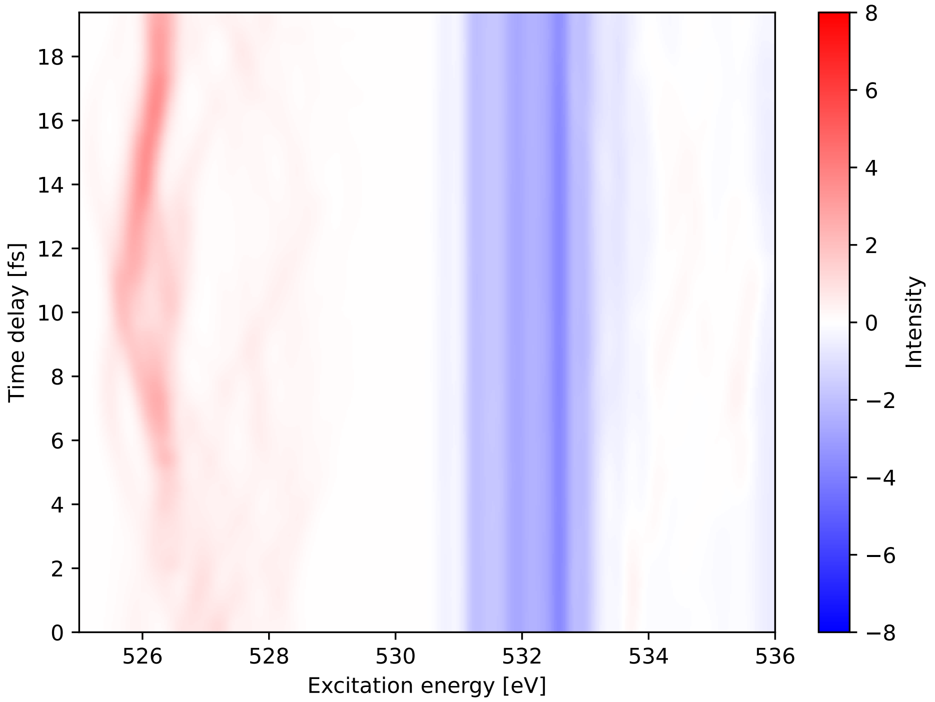

Suppl. Fig. 7 : Simulated thymine oxygen-edge X-ray absorption spectra for thymine for the first 800 au in time. Theoretical spectra are computed every 20 au and interpolated with Gaussians using `matplotlib`'s [4] `imshow`. For smoothing along the energy direction Gaussians with FWHM of 0.3 eV were used.

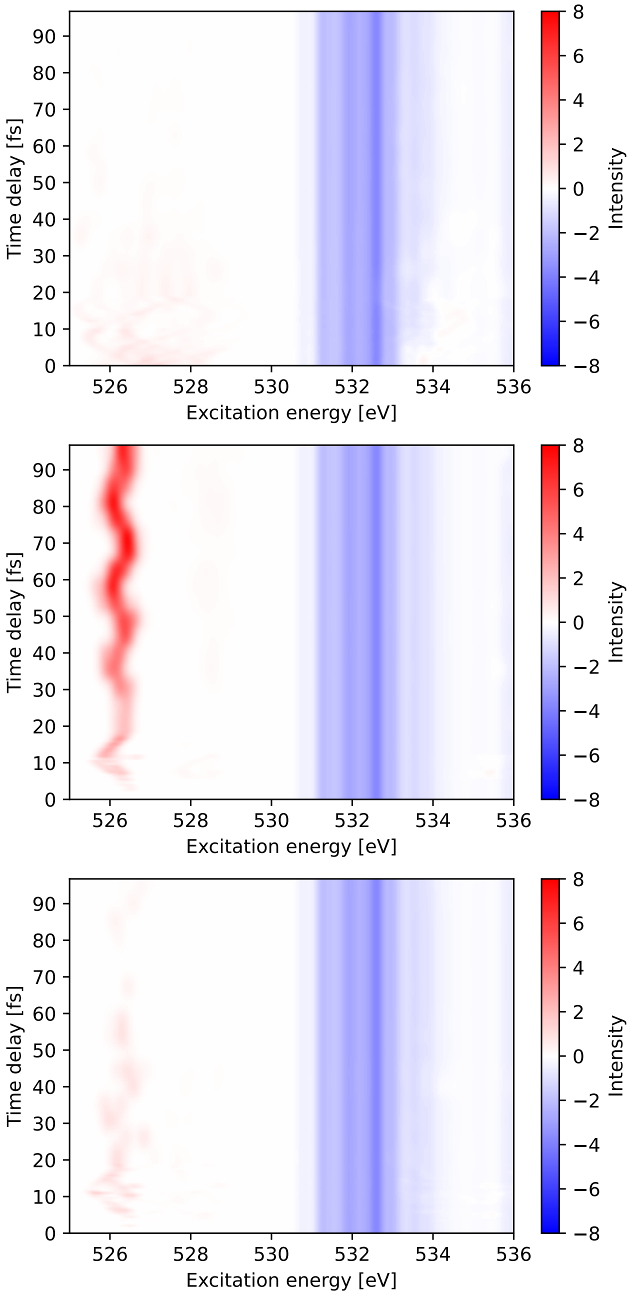

Suppl. Fig. 8 : Contribution from $\pi\pi^*$ (top) and $n\pi^*$ (center) states to the false color plot. The populated state was characterized by the sum of the oscillator strengths for the S_0 to S_1 and S_0 to S_2 transitions. $\pi\pi^*$ character was assumed if the sum was larger than 10 and and the populated state contributed more than 90% of the sum. $n\pi^*$ character if the populated state contributed 10% or less. Contributions that could neither be assigned to $\pi\pi^*$ nor $n\pi^*$ are plotted in the last panel.

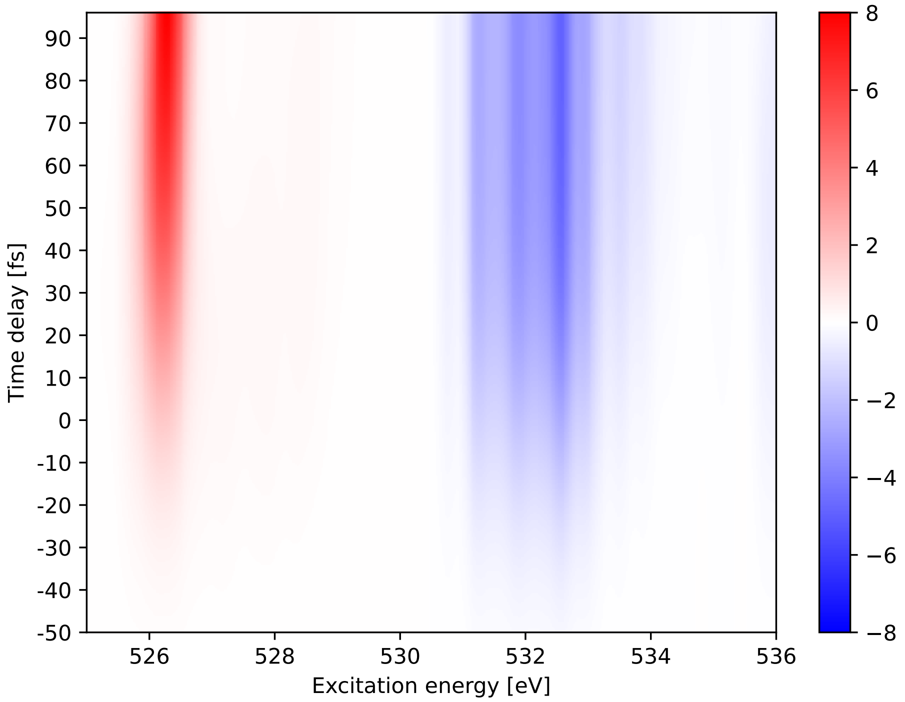

Suppl. Fig. 9 : False color plot with Gaussian smoothing using 70 fs FWHM and 0.3 eV FWHM. The absorption spectrum is computed in timesteps of 200 au (5 fs).

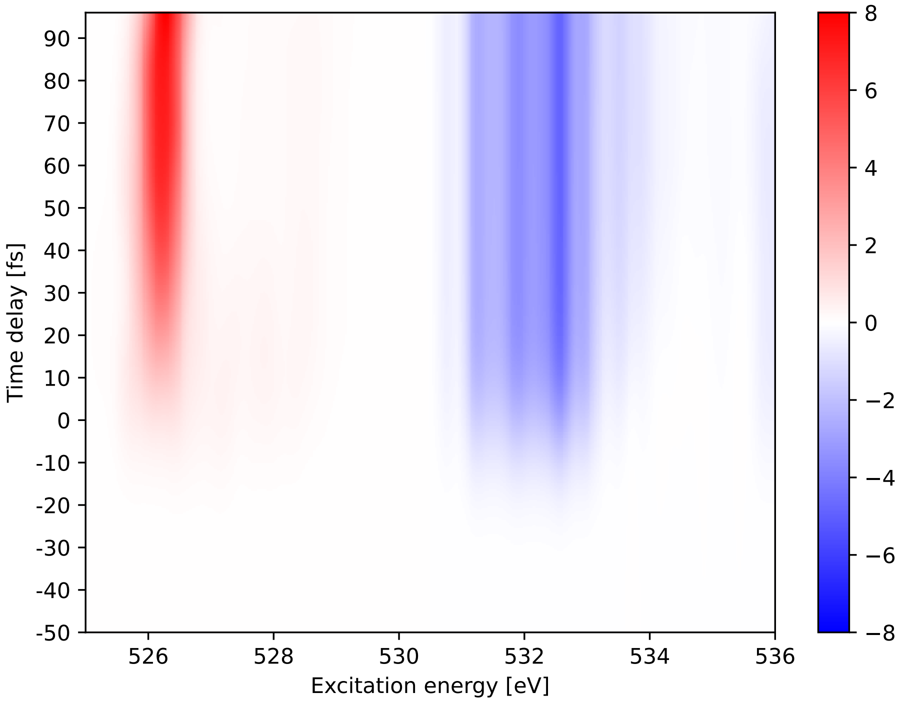

Suppl. Fig. 10 : False color plot with Gaussian smoothing using 30 fs FWHM and 0.3 eV FWHM. The absorption spectrum is computed in timesteps of 200 au (5 fs).

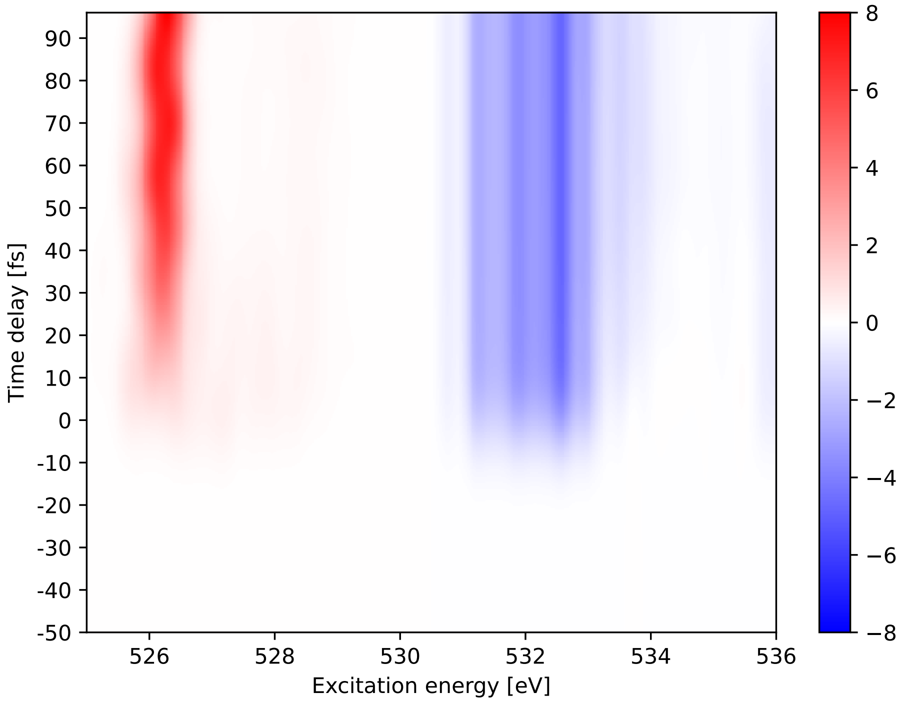

Suppl. Fig. 11 : False color plot with Gaussian smoothing using 20 fs FWHM and 0.3 eV FWHM. The absorption spectrum is computed in timesteps of 200 au (5 fs).

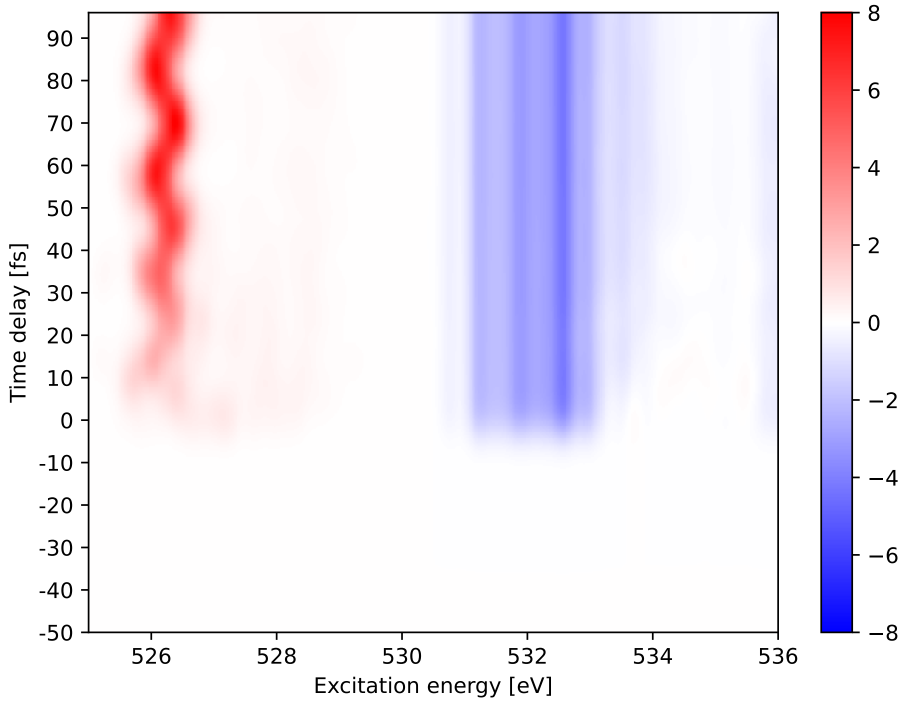

Suppl. Fig. 12 : False color plot with Gaussian smoothing using 10 fs FWHM and 0.3 eV FWHM. The absorption spectrum is computed in timesteps of 200 au (5 fs).

Suppl. Note 3

Initial dynamics for one initial condition with aug-cc-pVDZ

For one initial condition (IC 5), we have simulated its early dynamics from the Franck-Condon region to the first spawning event, comparing the time evolution obtained with cc-pVDZ and aug-cc-pVDZ. The time evolution is similar, as shown in Suppl. Fig. 13, both in terms of the time evolution of the C_5-C_6 and C_4-O_8 coordinates and the spawning event time (around 440 au). In this case, augmentation seems to play a minor role, as expected given the non-diffuse nature of the $n\pi^*$ and $\pi\pi^*$ states.

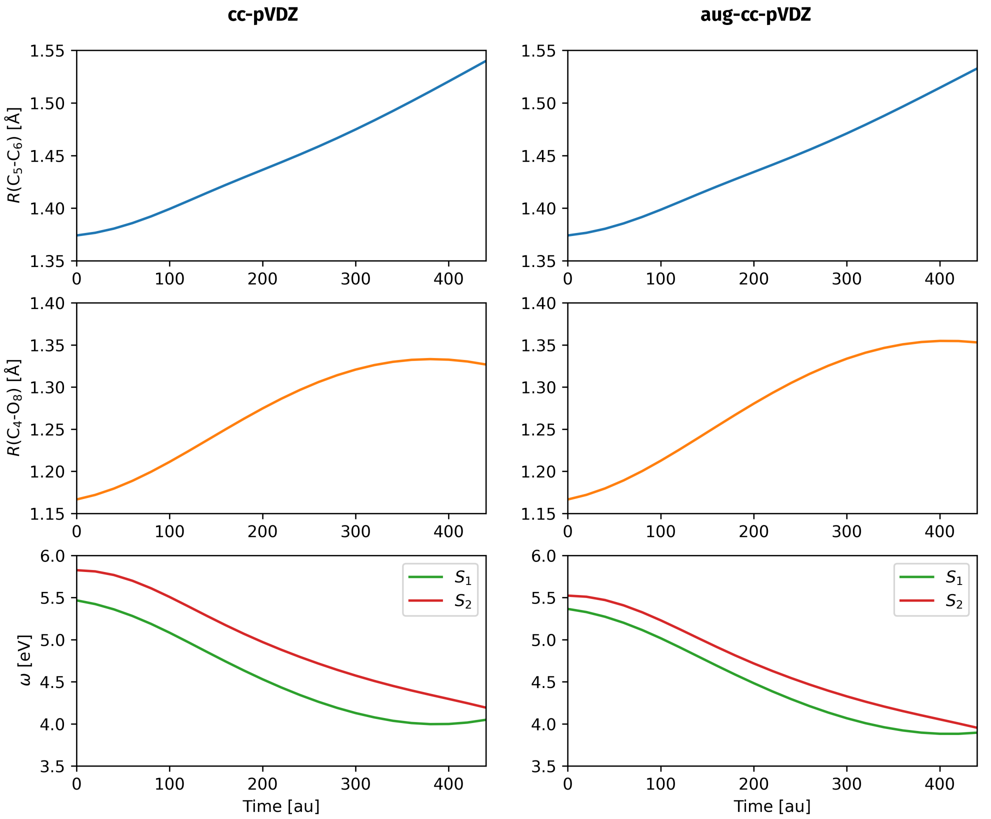

Suppl. Fig. 13 : Initial time evolution in C_5-C_6 and C_4-O_8 coordinates, and the associated excitation energies for S_1 and S_2, for IC 5 using cc-pVDZ (left) and aug-cc-pVDZ (right) in the AIMS/CCSD simulations.

Suppl. Note 4

Details on initial conditions that end in N-H dissociation

Two of the initial conditions are found to lead to N-H dissociation via a channel that involves a $\pi\sigma^*$ state. In Suppl. Fig. 14 and Suppl. Fig. 15 we track this process for one of these (IC 6). The other (IC 12) shows similar behavior. Suppl. Fig. 15 shows the energy of the first three adiabatic states along the parent TBF for IC6. We see that S_3 becomes nearly degenerate with S_2, and this changes the character of the latter from $\pi\pi^*$ to $\pi\sigma^*$, as can be seen from the NTOs shown in Suppl. Fig. 14. Later, S_2 becomes nearly degenerate with S_1 and the $\pi\sigma^*$ character is again transferred, now to S_1. The majority of the IC's population (74%) is on the $\pi\sigma^*$ state when it dissociates. The same is true of the other IC (68%). Since the ICs are terminated when dissociation occurs, we cannot determine whether the remaining population would also eventually lead to N-H dissociation. Assuming that the only dissociative events are the ones observed, we find a lower estimate for dissociative events in the simulation of 9%. We should emphasize, however, that this should be taken as a rough estimate, given the number of samples on which the estimate is based (our simulation only includes 16 ICs). More samples are needed to provide an accurate estimate of how common this channel is in the AIMS/CCSD/cc-pVDZ dynamics.

In particular, we note that the sample used in the dynamics has a larger proportion of very short N_1-H distances than in a larger sample consisting of 10000 geometries. In the 16 ICs we sampled, 13% has an N_1-H bond length of less than 0.90 Å and 31% of less than 0.95 Å, compared to 3% and 12% found in the larger sample, respectively. This suggests that the rough estimate of 9% for the dissociative $\pi\sigma^*$ pathway may be an overestimate, although actual dynamics simulations need to be run to decide what the precise percentage is.

Among the initial conditions specifically selected to have a short N-H bond length (less than 0.90 Å), which we here label as 5, 8, 30, 96, 131, 150, 165, 167, 177, 189, 191, 193, 370, 411, 495, 512, 530, we find that ICs 131, 177 (both with N_1-H bond lengths greater than 2.0 Å), and 96 ($>$ 1.8 Å), are dissociated and show $\pi\sigma^*$ character on a populated TBF. IC 5, 189, and 411 have $\pi\sigma^*$ character on a populated TBF with an N_1-H bond length of 1.5 Å, 1.274 Å, and 1.531 Å, respectively. In IC 512, one of the TBFs is dissociating (N_1-H bond length 1.753 Å) but does not have a significant population. In several cases, these ICs encounter numerical issues and the simulation of these ICs has therefore not been completed. In the remaining ICs, we did not find any involvement of the $\pi\sigma^*$ state. Figure Suppl. Fig. 16 shows the plot in Figure 5D, where we have also included the ICs specifically chosen with short initial N_1-H. As in Figure 5D, we see that the dissociating ICs spawn at large N_1-H bond lengths.

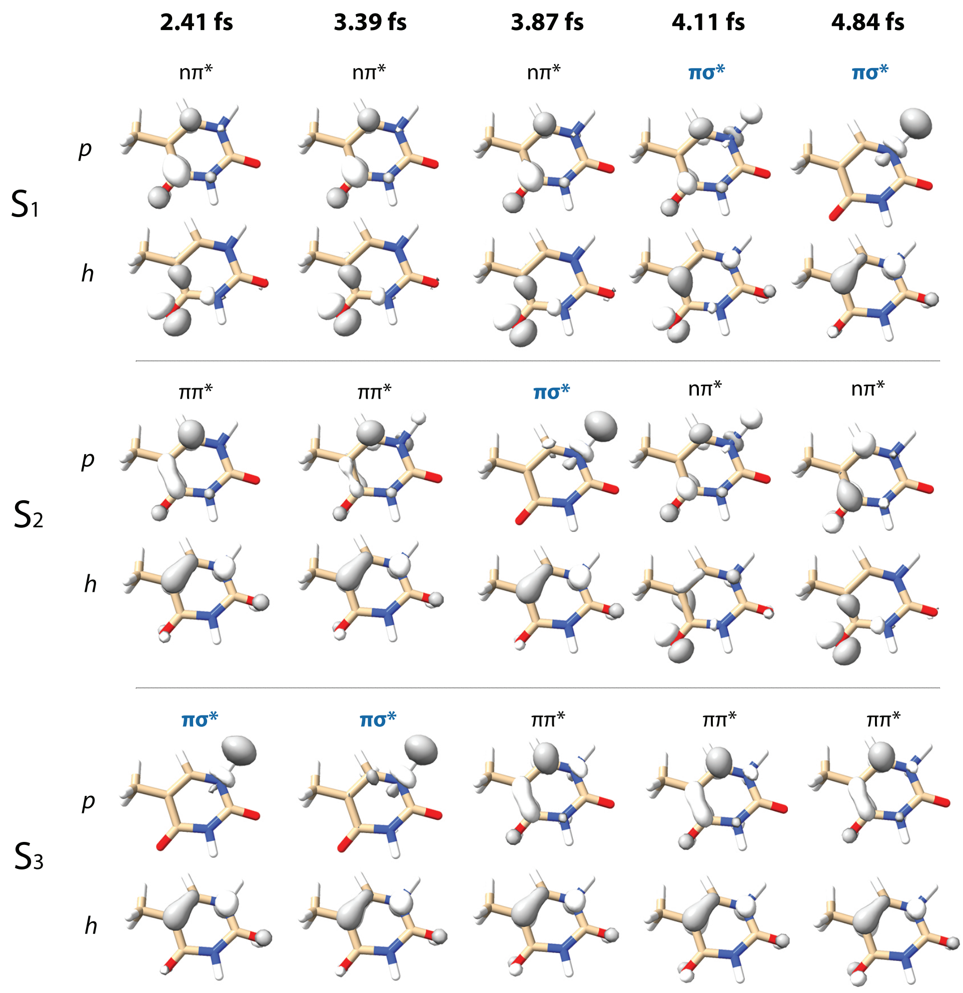

Suppl. Fig. 14 : Principal NTOs (particle denoted by p, hole by h) for S_1, S_2, and S_3 for IC 6, from 2.41 to 4.84 fs (100 to 200 au) At 2.41 fs S_1 has primarily $n\pi^*$ character, S_2 primarily $\pi\pi^*$, and S_3 is $\pi\sigma^*$. As the gap between S_2 and S_3 narrows, S_2 begins to assume more $\pi\sigma^*$ character, and by 3.87 fs , the principal NTO of S_2 has $\pi\sigma^*$ character.

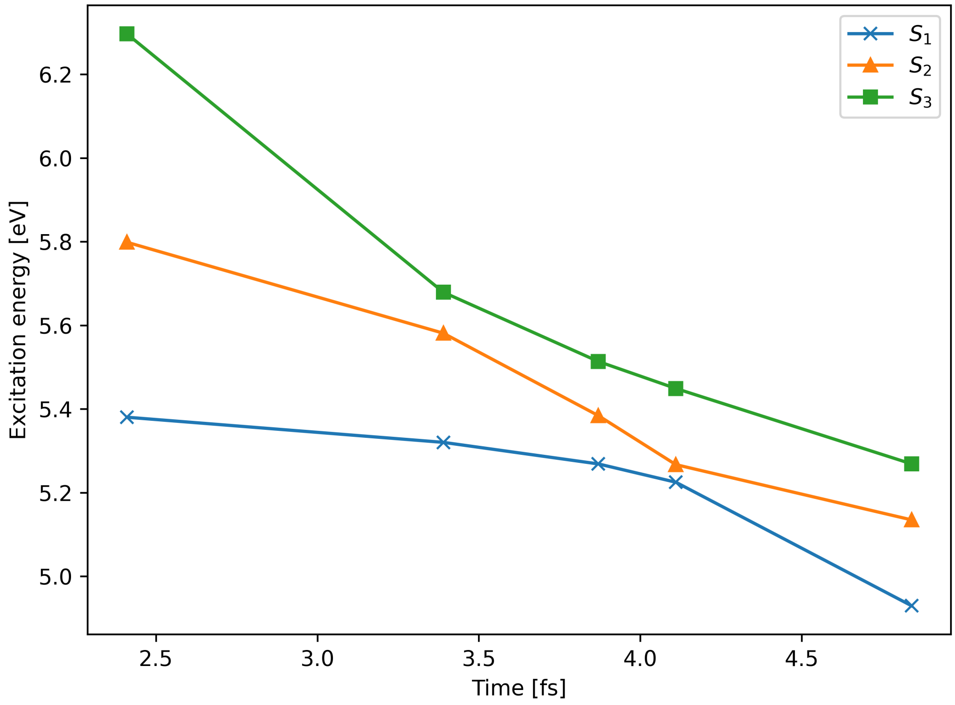

Suppl. Fig. 15 : Excitation energies for the first three excited states for IC 6 at 2.41, 3.39, 3.87, 4.11, and 4.84 fs.

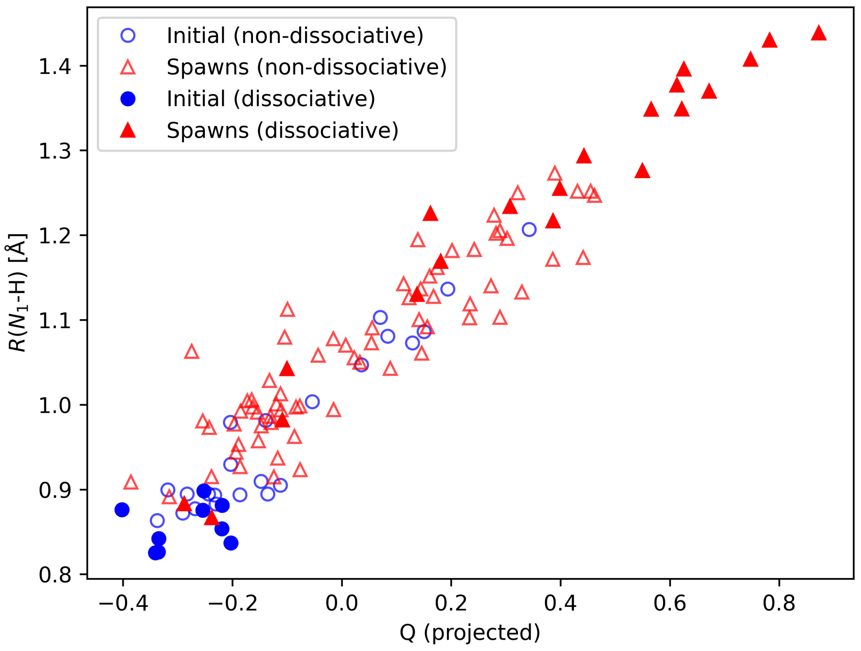

Suppl. Fig. 16 : Initial geometries and spawning geometries, in terms of the N_1-H bond length and the projected value for the N_1-H bond stretch normal mode, for the set of initial conditions used in the dynamics as well as the conditions specifically chosen to have short initial N_1-H bond lengths (less than 0.9 Å). Displacements Q along this the N_1-H bond stretch normal mode are given in Bohr. Dissociating initial conditions appear to spawn at higher N_1-H bond lengths than non-dissociating conditions. This plot can be compared to Figure 5D, which only includes the initial conditions used in the simulation.

Suppl. Note 5

Simulated UV-vis absorption spectrum

The UV-vis absorption spectrum was calculated at the CCSD/aug-cc-pVDZ+KBJ(3-4) and CC3/aug-cc-pVDZ+KBJ(3-4) level for the ground state equilibrium geometry, determined with CCSD/aug-cc-pVDZ. To improve the description of possible Rydberg states in the spectrum, a set of Rydberg-type functions with quantum numbers $n = \{3, 3.5, 4\}$, generated according to the prescription of Kaufmann, Baumeister and Jungen [5], was added. These additional functions are denoted "KBJ(n=3–4)". Simulated spectra are shown in Suppl. Fig. 17. In the case of CCSD, we have additionally simulated the UV-vis absorption spectrum obtained by averaging over the 16 ICs in the Wigner sample (see upper panel, Suppl. Fig. 17). Finally, to interpret the CC3 spectrum in Suppl. Fig. 17, NTOs for the bright CC3/aug-cc-pVDZ+KBJ(3-4) excitations are shown in Suppl. Table 1.

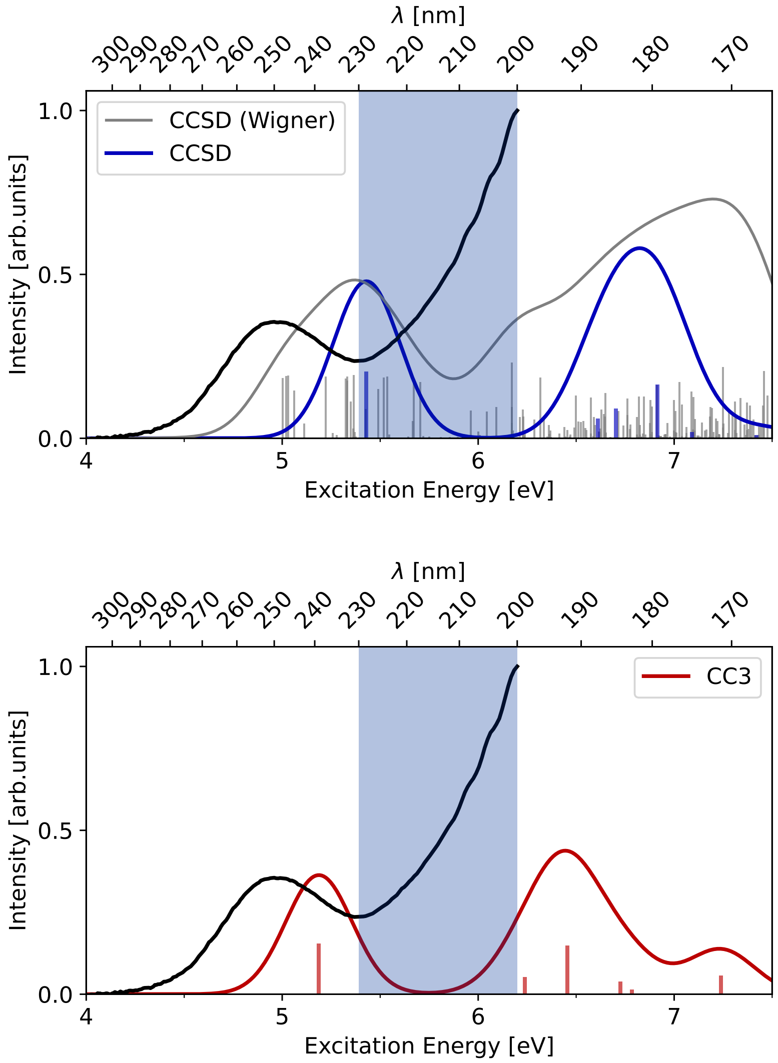

Suppl. Fig. 17 : The absorption spectrum of thymine calculated at the CCSD and CC3 level with aug-cc-pVDZ+KBJ(3-4) basis set at the ground state equilibrium geometry. In the case of CCSD (upper panel), we have also calculated the absorption spectrum obtained by averaging over the 16 initial conditions sampled from the Wigner distribution. The black curve represents the experimental gas-phase spectrum. The wavelength range between 230 nm and 200 nm is highlighted in blue. Individual excitations (for both Wigner and Franck-Condon spectra) were broadened with Gaussian functions of 0.4 eV FWHM and no shift was applied. Intensities are given in arbitrary units and are scaled.

Suppl. Table 1 : Excitation energies (ω), oscillator strengths (f) and NTOs for the bright CC3/aug-cc-pVDZ+KBJ(3-4) excitations of thymine. An iso value of 0.03 electrons/bohr^3 was used.

	ω [eV]	$f \times 100$	hole NTO	particle NTO
S_2	5.19	15.47	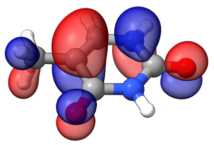	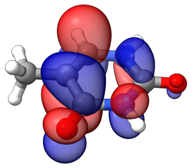
S_4	6.24	5.20	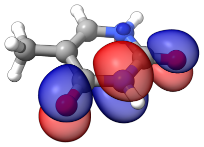	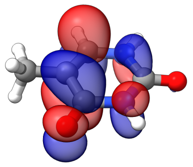
S_7	6.46	14.91	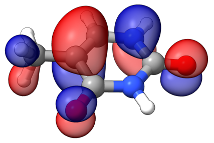	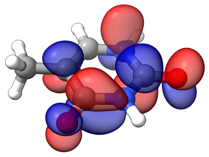
S_9	6.73	3.91	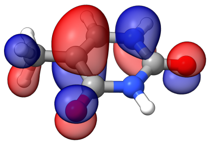	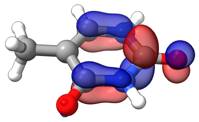
S_{11}	6.78	1.43	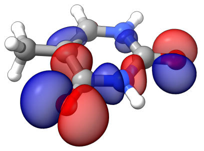	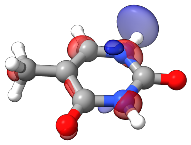
S_{15}	7.24	5.75	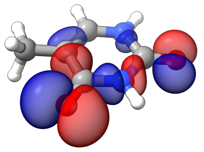	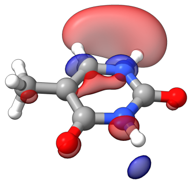

Suppl. Note 6

Inclusion of S_3 in dissociative initial conditions

The dissociative pathway involves a near-degeneracy between S_2 and S_3 due to the $\pi\sigma^*$ state lowering in energy along the NH bond stretching coordinate. We have therefore investigated the effect of including S_3 explicitly in the dynamics simulations by re-running the two initial conditions that follow the $\pi\sigma^*$ pathway, where we now include S_3. We do not observe any significant changes when including S_3. The trajectories that follow the dissociative pathway acquire the same population. There is thus no suppression of the pathway by inclusion of S_3. Figure Suppl. Fig. 18 shows the NH bond lengths for the trajectory basis functions spawned from IC 6 and 12. Inclusion of S_3 leads to one additional TBF (see red curve, lower left) which acquires a 3% population in the case of IC 6. No population transfer to S_3 is observed for IC 12. Note that the first spawning event (which leads to dissociation, orange curve) is identical with and without S_3 for both ICs and results in the same transfer of population from S_2 to S_1.

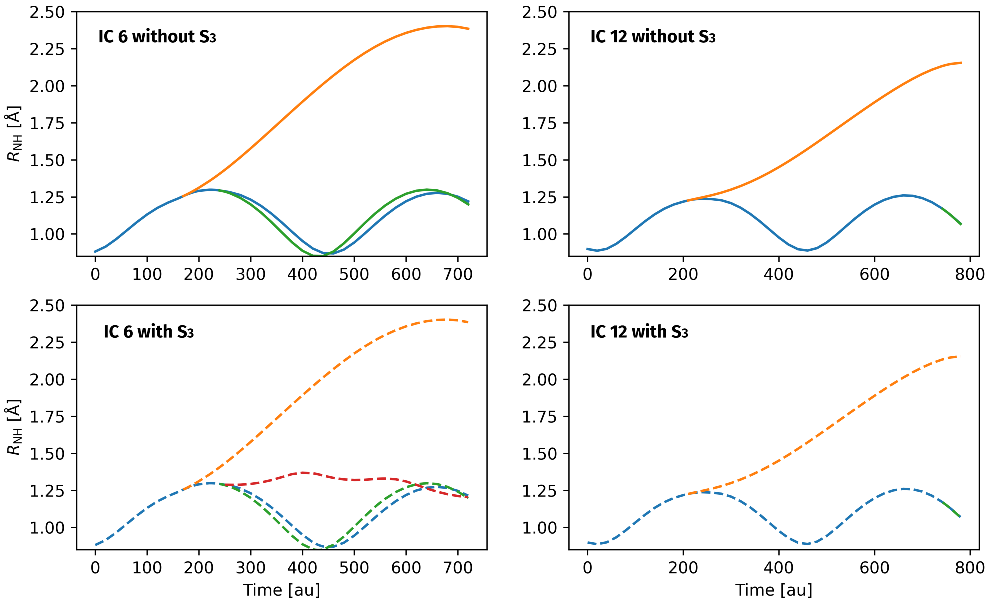

Suppl. Fig. 18 : NH bond lengths R_{NH} versus time for dissociative initial conditions without (upper panels, solid) and with (lower panels, dashed) S_3 in the AIMS dynamics simulations. The initial conditions are given in blue and spawned trajectory basis functions in orange, green, and red.

Suppl. Note 7

Potential energy curves along the NH bond with CC3 and aug-cc-pVDZ

In Suppl. Fig. 19, we provide the low-lying electronic energies determined using CCSD/cc-pVDZ and CC3/cc- pVDZ. We note that the low-lying states S_1, S_2, and S_3 show similar behavior for both CCSD and CC3, with similar energy gaps and a similar crossing region (at $Q = 0.5$) for the $\pi\sigma^*$ state. It appears that S_1 becomes degenerate with the ground state with CC3 somewhat earlier than with CCSD (compare S_0/S_1 gap at $Q = 2.0$). Higher-lying states show more pronounced differences, but these are not expected to have an impact on the dynamics starting from the $\pi\pi^*$ state.

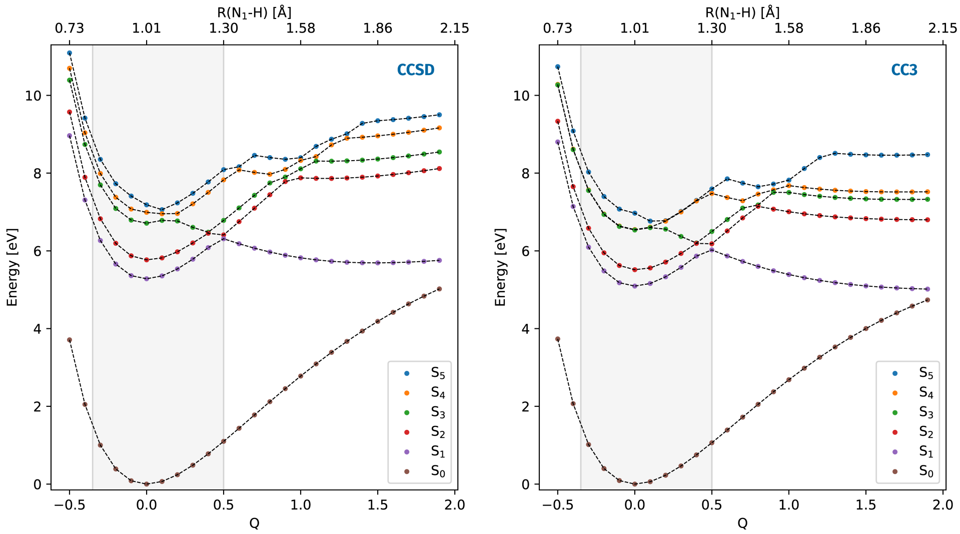

Suppl. Fig. 19 : Potential energy curves determined using CCSD/cc-pVDZ (left) and CC3/cc-pVDZ (right) along the N-H stretching coordinate Q. The shaded area corresponds to the region where spawning occurs in the AIMS simulations (for more details, see the main text).

In Suppl. Fig. 20, we show the same curves calculated using CCSD/cc-pVDZ and CCSD/aug-cc-pVDZ. We first note that S_3 is stabilized in the Franck-Condon region with aug-cc-pVDZ. This is expected due to the π-3s Rydberg character of the dissociative $\pi\sigma^*$ state close to the Franck-Condon geometry. This diffuse character decreases along the NH coordinate, as also observed in other systems [6]. As a result, the $\pi\sigma^*$ state is similarly described for longer NH bond lengths, which is also the part of the potential energy curves relevant to the simulated dynamics starting from S_2 and interacting with S_3 only for longer NH bond lengths. At $Q \approx 0.5$, we see that the

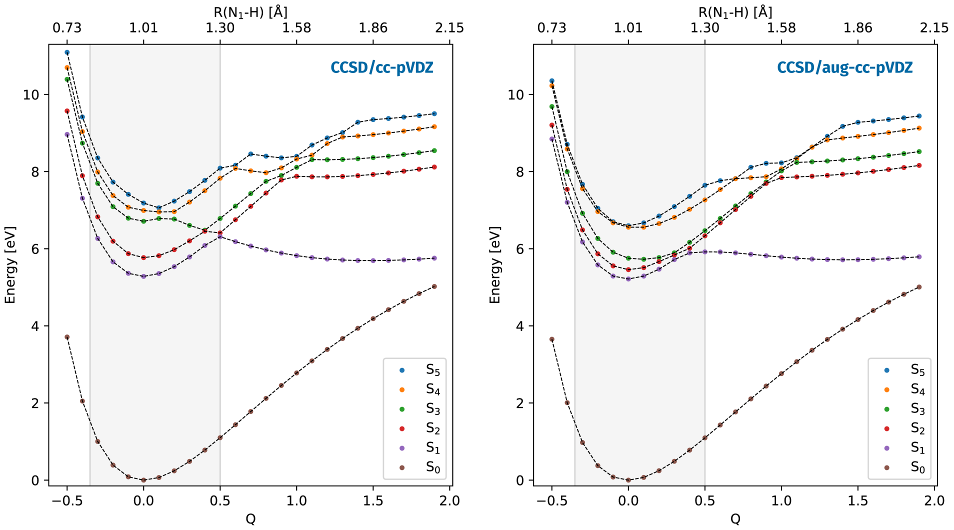

Suppl. Fig. 20 : Potential energy curves determined using CCSD/cc-pVDZ (left) and CCSD/aug-cc-pVDZ (right) along the N-H stretching coordinate Q. The shaded area corresponds to the region where spawning occurs in the AIMS simulations (for more details, see the main text).

dissociative $\pi\sigma^*$ state appears to be accessible at somewhat shorter NH bond lengths ($Q \approx 0.4$) with aug-cc-pVDZ compared to cc-pVDZ.

Suppl. Note 8

Nitrogen-edge spectrum at Franck-Condon, at the $n\pi^*$ minimum, and at $Q = 1.0$ in the dissociative N_1-H pathway

In Suppl. Fig. 21, we give simulated nitrogen-edge X-ray absorption spectra at three geometries relevant to the dynamics: the ground state minimum, the $n\pi^*$ minimum, and a displaced geometry along the N_1-H dissociation pathway (in particular, $Q = 1.0$ for the normal mode associated with the bond stretch, corresponding to an N_1-H bond length of about 1.6 Å). Some observations can be made. First, the $n\pi^*$ and $\pi\pi^*$ signals are located at around 396–397 eV, with the $\pi\pi^*$ signal being the brighter of the two. We therefore expect a signal at 396–397 eV which mostly disappears in the first 50 fs as the wavepacket undergoes the $\pi\pi^*$ to $n\pi^*$ transition. At the extended N_1-H geometry ($Q = 1.0$), the $S_1(\pi\sigma^*)$ state has a signal that is shifted down to around 395 eV, although the exact location of this peak is expected to move as the hydrogen atom dissociates. Furthermore, a ground state peak is shifting down significantly, to around 398 eV, because the gap between the ground state and $\pi\sigma^*$ state is becoming progressively smaller as the N_1-H bond is extended. If some of the population transfers to the ground state, this peak could also be visible in a nitrogen-edge spectrum.

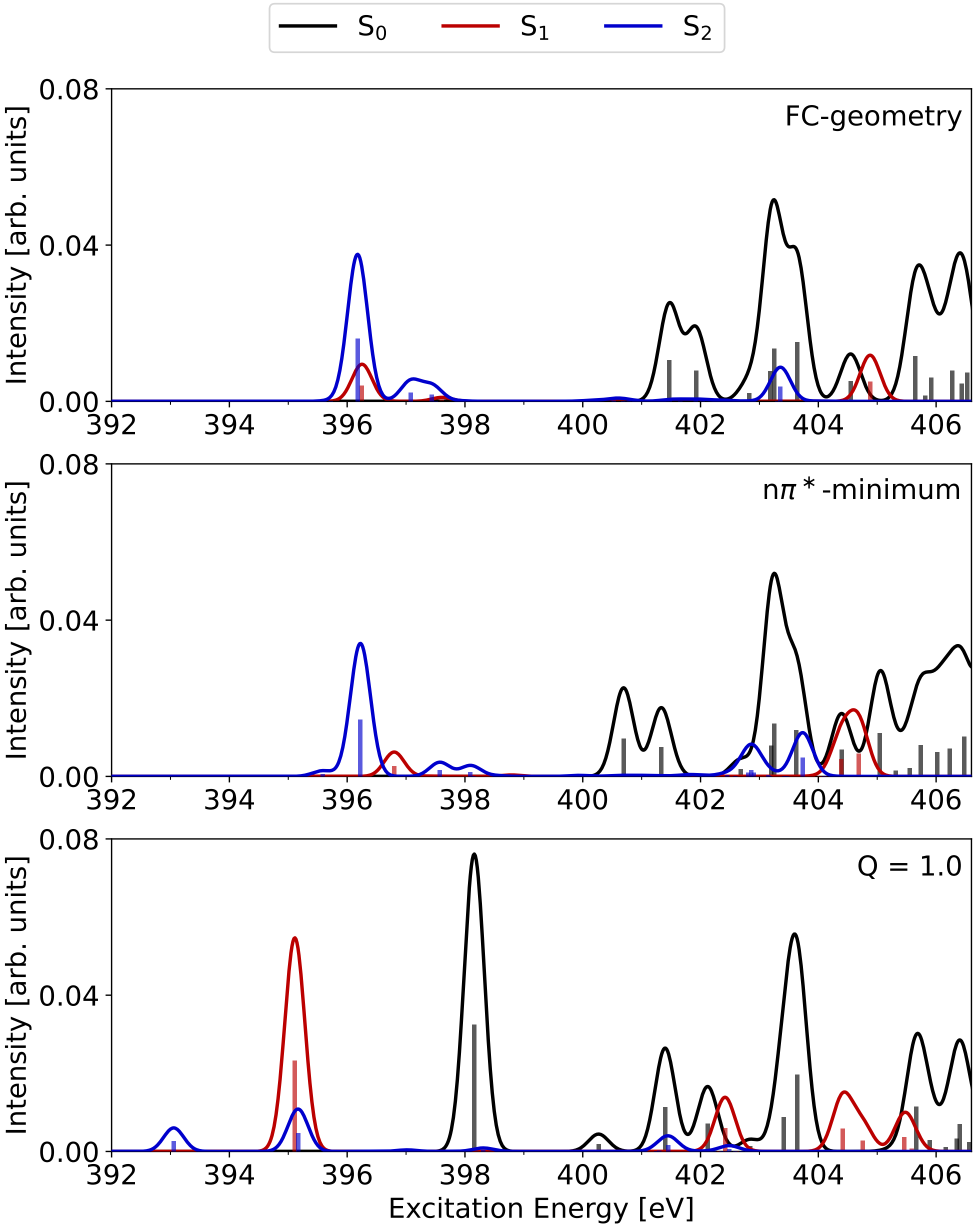

Suppl. Fig. 21 : Nitrogen-edge X-ray absorption spectrum at the Franck-Condon point (FC-geometry), in the $n\pi^*$ minimum, and for a displacement $Q = 1.0$ along the N_1-H stretching mode. The spectra were calculated with CC3/cc-pVDZ for the core excited states and CCSD/cc-pVDZ for the ground state and the valence excited states. Individual excitations were broadened with Gaussian functions of 0.4 eV FWHM and all spectra were shifted by −1.02 eV to align with the experiment reported in Ref. 7.

Suppl. Note 9

Wigner sampling: selection of initial conditions

The initial conditions (positions and momenta) used in the 16 AIMS runs were sampled from a harmonic Wigner distribution based on the ground state equilibrium geometry, determined at the CCSD/aug-cc-pVDZ level. The converged ground state geometry, along with the harmonic frequencies, are given in Suppl. Table 2. The harmonic frequencies were determined by evaluating the molecular Hessian, which was performed with e^T using central differences (dx=10^{-4} a_0) on the analytical CCSD gradients evaluated at displacements from the equilibrium geometry. The Hessian was used to generate the Wigner samples, which we have numbered as ICs 5–20. ICs 0–4 were used in initial tests to debug the interface and were not used in the dynamics simulations. For reference, ICs 6 and 12 lead to N-H dissociation, while 7 and 14 were re-run with SCCSD because they terminate in complex energy regions when run with CCSD. Positions and momenta of the individual samples are also available [8].

Suppl. Table 2 : Ground state equilibrium geometry (in Å) and vibrational harmonic frequencies (in cm^{-1}) at the CCSD/aug-cc-pVDZ level.

C	1.630701110258	−0.071137104515	−0.000217015039	106	536	906	1414	1812
C	−0.209693539530	1.539822276693	0.000058364321	144	548	977	1424	3052
C	−0.761020102709	−0.825618775384	0.000090263142	174	607	1027	1449	3126
C	−1.179422230038	0.591016860409	0.000089892193	276	680	1070	1469	3139
C	−2.656435643450	0.893598512239	0.000225801361	292	739	1170	1494	3235
N	0.627861839137	−1.031930751046	−0.000192591426	392	763	1218	1524	3629
N	1.145275760481	1.226181730571	−0.000168258378	392	780	1268	1739	3676
O	2.822138135024	−0.341269451406	−0.000301832749	460	811	1397	1783	
O	−1.528917478290	−1.778211641752	0.000340887262					
H	1.848723077280	1.951968102483	−0.000249352114					
H	−0.437664838893	2.607440739349	0.000178809529					
H	0.943714834308	−1.996305266184	−0.000110287090					
H	−2.829665811383	1.981197603193	−0.000029746644					
H	−3.140793068686	0.454894321068	0.887377393240					
H	−3.141002043508	0.454452844282	−0.886592327609					

Suppl. Note 10

Spawning geometries and the accessibility of the conical intersection seam along the C_5-C_6 stretching coordinate

In Suppl. Fig. 22, we plot the spawning geometries in terms of their C_5–C_6 and C_4–O_8 bond lengths. Here, "spawning geometries" refers to geometries where new basis functions (TBFs) are created, which indicates where we may see a significant transfer of population between the states. The distribution of spawning geometries shows that the seam is accessible along a C_5–C_6 lengthening coordinate that connects the Franck-Condon point (S_0^{min}) and the minimum-energy conical intersection (S_1/S_2 MECI). All but two of the spawns correspond to transfer from S_2 to S_1.

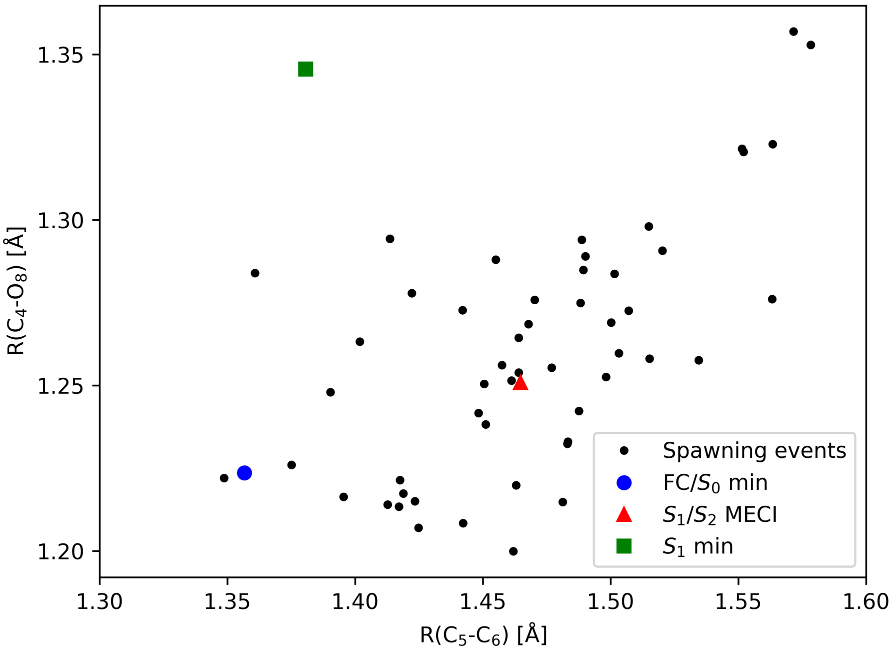

Suppl. Fig. 22 : Spawning geometries in the C_5–C_6 and C_4–O_8 bond coordinates. Black dots correspond to spawning geometries. The wavepacket accesses the S_1/S_2 MECI conical intersection seam primarily along a C_5-C_6 elongation from the initial Franck-Condon region, with a cluster of spawning events occurring close to the minimum energy conical intersection (S_1/S_2 MECI).

Suppl. Note 11

Stationary points on the excited state surfaces: the minimum energy S_1/S_2 conical intersection and the S_1 minimum

To interpret the wavepacket dynamics, we also located stationary points on the excited state surfaces. In Suppl. Table 3 and Suppl. Table 4, we list the geometry for the $n\pi^*$ minimum and the minimum energy conical intersection between the S_1 and S_2 states, respectively. These stationary points were determined at the same level as the dynamics (CCSD/cc-pVDZ).

Suppl. Table 3 : $S_1(n\pi^*)$ minimum geometry (in Å) and vibrational harmonic frequencies (in cm^{-1}) at the CCSD/cc-pVDZ level.

C	1.626856184467	−0.090172437156	0.013282935761	90	460	801	1404	1869
C	−0.197850164697	1.572388519191	−0.033300408283	102	467	948	1428	3069
C	−0.740709833470	−0.739313238235	−0.079521724593	104	498	1015	1446	3141
C	−1.176243220430	0.598835299691	−0.065959144242	215	527	1064	1482	3169
C	−2.652953867469	0.918586250733	−0.094848197888	249	582	1155	1494	3278
N	0.630784739942	−1.058855452370	−0.174427947644	257	605	1220	1499	3651
N	1.164641231138	1.205510451408	−0.019672119665	340	752	1261	1544	3698
O	2.794813959974	−0.394624876859	0.158035521208	396	760	1296	1633	
O	−1.546761142159	−1.810516595404	−0.194583290615					
H	1.881303447604	1.906943526328	0.115985843483					
H	−0.412611083215	2.640305248447	−0.000321107921					
H	0.924394117785	−1.993233441610	0.093943333488					
H	−2.811463058403	2.006697839314	−0.031363123742					
H	−3.180294992549	0.445499759315	0.751943675010					
H	−3.121321727307	0.554762000579	−1.026259737912					

Suppl. Table 4 : S_1/S_2 minimum energy conical intersection geometry (in Å) at the CCSD/cc-pVDZ level

C	1.620884204303	−0.107517382267	−0.042077345164
C	−0.175634683514	1.618168806981	−0.079419662615
C	−0.778255418212	−0.815707290539	−0.050927310541
C	−1.173750723137	0.548693928425	−0.059187433420
C	−2.634883093476	0.878862929277	−0.041363924381
N	0.652954617850	−1.060239304783	−0.061438342476
N	1.128940987061	1.228004642700	−0.030710272580
O	2.828783658498	−0.280358835324	−0.019138916975
O	−1.519845543610	−1.823734952390	−0.033898210074
H	1.880975271175	1.913878716948	0.019668160698
H	−0.396377317533	2.680221014786	−0.171050605554
H	0.948152824540	−2.027583594230	−0.121911512159
H	−2.799214915932	1.965091087367	0.048680697186
H	−3.133549718077	0.370648144289	0.805126231045
H	−3.144410128255	0.521765471910	−0.956125307028

Suppl. Note 12

Interface for AIMS dynamics with CC theory

The AIMS simulations were performed with the FMS program developed in the Todd J. Martínez group. Since the CCSD and SCCSD nuclear gradients and nonadiabatic coupling elements were implemented in the e^T program [2], we have written an interface that connects FMS with e^T. Over the course of the simulation, FMS requests information from the selected electronic structure program, which, in our case, is e^T. FMS requests the nuclear gradients and derivative coupling elements evaluated at the centers of TBFs and at the centroids between pairs of TBFs. This is all the information required to propagate the nuclear wavefunction in AIMS [9, 10].

To easily retrieve this information, we implemented a few objects/routines that allow FMS (and other programs) to use e^T as a library. In particular: routines to set up and finalize e^T, and drivers to compute gradients and couplings. The changes to e^T are planned to be made publicly available in an upcoming release of the program. On the FMS side, the interface template for electronic structure programs already exists and has been copied. In FMS, we have added a new model/electronic structure program (e^T) and implemented the routine that will be invoked for this new model. In particular, FMS calls this routine when it requires new electronic structure information, passing to it a trajectory object (including the geometry), a specification of the states (i, j), and whether or not to compute the coupling between them. The routine then invokes an e^T driver to compute gradients and couplings and populates the FMS trajectory with the required data.

We need to make sure that the coupling elements in FMS have a consistent phase. For this we use a simple orbital phasing procedure. The trajectory objects in FMS store the previous orbital coefficients and excited state amplitudes. In each new timestep, we pass this information on to e^T where the current orbitals are phased. The current and previous orbitals are compared by evaluating the matrix

$$A_{pq}(\boldsymbol{x}) = \sum_{\alpha\beta} C_{\alpha p}(\boldsymbol{x}_0) S_{\alpha\beta}(\boldsymbol{x}) C_{\beta q}(\boldsymbol{x}), \tag{14}$$

where α, β denotes atomic orbital indices and p, q denotes molecular orbital indices. The orbital coefficients are denoted $C_{\alpha p}$ and the atomic orbital overlap matrix as $S_{\alpha\beta}$. Finally, $\boldsymbol{x}_0$ refers to the previous geometry and $\boldsymbol{x}$ to the current geometry.

If an orbital flips sign from $\boldsymbol{x}_0$ to $\boldsymbol{x}$, the corresponding diagonal term of $\boldsymbol{A}$ will be approximately -1. If two orbitals change order energetically, then the corresponding 2-by-2 block will be dominated by the off-diagonal components. We first look for orbitals that change their order, assuming that the orbital has changed order energetically if

$$|A_{ij}| + |A_{ji}| > |A_{ii}| + |A_{jj}|. \tag{15}$$

After this procedure finishes, the diagonal components of $\boldsymbol{A}$ will have numbers close to 1 or -1 on the diagonal and small off-diagonal components. If the diagonal element is -1, we change the sign of the corresponding orbital. In this way, we obtain a consistent

phase for the orbitals, which allows us to compare current and previous excited state amplitudes consistently by evaluating the state overlap matrix

$$S_{ij}(\boldsymbol{x}) = \boldsymbol{R}_i(\boldsymbol{x}_0)^T \boldsymbol{R}_j(\boldsymbol{x}). \tag{16}$$

If the diagonal is close to -1, we flip the sign of the excited state vector $\boldsymbol{R}_i(\boldsymbol{x})$. This should ensure a consistent phase for the coupling. The $\boldsymbol{S}$ matrix is also used to detect intersection jumps. If the off-diagonal elements dominate, where we again use the condition in Equation (15), then FMS will reject the timestep (provided that the state that flips is important for the dynamics) and attempt a shorter timestep.

Supplementary References

[1] Wolf, T. J. A. *et al.* Probing ultrafast $\pi\pi^*/n\pi^*$ internal conversion in organic chromophores via K-edge resonant absorption. *Nat. Commun.* **8**, 29 (2017).

[2] Folkestad, S. D. *et al.* eT 1.0: An open source electronic structure program with emphasis on coupled cluster and multilevel methods. *J. Chem. Phys.* **152**, 184103 (2020).

[3] Coriani, S. & Koch, H. Communication: X-ray absorption spectra and core-ionization potentials within a core-valence separated coupled cluster framework. *J. Chem. Phys.* **143**, 181103 (2015).

[4] Hunter, J. D. Matplotlib: A 2d graphics environment. *Computing in Science & Engineering* **9**, 90–95 (2007).

[5] Kaufmann, K., Baumeister, W. & Jungen, M. Universal Gaussian basis sets for an optimum representation of Rydberg and continuum wavefunctions. *J. Phys. B: Atom. Mol. Opt. Phys.* **22**, 2223–2240 (1989).

[6] Roberts, G. M. & Stavros, V. G. The role of $\pi\sigma^*$ states in the photochemistry of heteroaromatic biomolecules and their subunits: insights from gas-phase femtosecond spectroscopy. *Chem. Sci.* **5**, 1698–1722 (2014).

[7] Plekan, O. *et al.* A theoretical and experimental study of the near edge X-ray absorption fine structure (NEXAFS) and X-ray photoelectron spectra (XPS) of nucleobases: Thymine and adenine. *Chem. Phys.* **347**, 360–375 (2008). Ultrafast Photoinduced Processes in Polyatomic Molecules.

[8] Kjønstad, E. F. *et al.* Data for "photoinduced hydrogen dissociation in thymine predicted by coupled cluster theory" (10.5281/zenodo.10733950) (2024).

[9] Ben-Nun, M., Quenneville, J. & Martínez, T. J. Ab initio multiple spawning: Photochemistry from first principles quantum molecular dynamics. *J. Phys. Chem. A* **104**, 5161–5175 (2000).

[10] Ben-Nun, M. & Martínez, T. J. Ab initio quantum molecular dynamics. *Adv. Chem. Phys.* **121**, 439–512 (2002).
